# A Comparative Study between Spanish and British SARS-CoV-2 Variants

**DOI:** 10.3390/cimb43030140

**Published:** 2021-11-16

**Authors:** Jose A. Jimenez Ruiz, Cecilia Lopez Ramirez, Jose Luis Lopez-Campos

**Affiliations:** 1 Research Group on Electronic Technology and Industrial Computing (TIC-150), Department of Artificial Intelligence, University of Seville, 41013 Sevilla, Spain; jantonio.jimenez@gmail.com; 2 Unidad Médico-Quirúrgica de Enfermedades Respiratorias, Instituto de Biomedicina de Sevilla (IBiS), Hospital Universitario Virgen del Rocío/Universidad de Sevilla, 41013 Sevilla, Spain; ceclopram@gmail.com; 3 Centro de Investigación Biomédica en Red de Enfermedades Respiratorias (CIBERES), Instituto de Salud Carlos III, 28029 Madrid, Spain

**Keywords:** COVID-19, SARS-CoV-2, genomics characterization, computational docking, in silico

## Abstract

The study of the interaction between the SARS-CoV-2 spike protein and the angiotensin-converting enzyme 2 (ACE2) receptor is key to understanding binding affinity and stability. In the present report, we sought to investigate the differences between two already sequenced genome variants (Spanish and British) of SARS-CoV-2. **Methods:** In silico model evaluating the homology, identity and similarity in the genome sequence and the structure and alignment of the predictive spike by computational docking methods. **Results:** The identity results between the Spanish and British variants of the Spike protein were 28.67%. This close correspondence in the results between the Spanish and British SARS-CoV-2 variants shows that they are very similar (99.99%). The alignment obtained results in four deletions. There were 23 nucleotide substitutions also predicted which could affect the functionality of the proteins produced from this sequence. The interaction between the binding receptor domain from the spike protein and the ACE2 receptor produces some of the mutations found and, therefore, the energy of this ligand varies. However, the estimated antigenicity of the British variant is higher than its Spanish counterpart. **Conclusions:** Our results indicate that minimal mutations could interfere in the infectivity of the virus due to changes in the fitness between host cell recognition and interaction proteins. In particular, the N501Y substitution, situated in the RBD of the spike of the British variant, might be the reason for its extraordinary infective potential.

## 1. Introduction

The ongoing pandemic of coronavirus disease 2019 (COVID-19) that started in December 2019, in Wuhan, China, and its most widely feared respiratory complication, severe acute respiratory syndrome, has struck society hard from a medical, economic and social perspective [1]. To make matters worse, the impact of the pandemic has been aggravated by the appearance of successive variants of the virus: this has had different consequences, generating considerable uncertainty in both society and the health services. As a result, in order to fight the disease, it has become a high priority to fully understand the variants which have already been detected, as well as to identify promptly the new variants [2]. Specifically, the variations in the membrane proteins which mediate the entry of the virus into the cell are thought to play a relevant role in the infective process and have therefore attracted the attention of the scientific community.

The SARS-CoV-2 genome consists of a single positive-sense RNA strand (29.8 kb–29.9 kb) that encodes three external structural proteins: the membrane, the envelope and spike proteins [3], of which the spike protein is responsible for initiating the infection by binding to angiotensin-converting enzyme 2 (ACE2) receptors on the cell membrane, thus initiating the process which allows the virus to enter the cell [4]. Accordingly, the study of the interaction between the spike protein and the ACE2 receptor is a key step to understanding binding affinity and stability [5].

Some previous works have evaluated the differences between different variants of the virus and have identified some relevant mutations using in vitro models and in silico models [6,7,8]. In the present report, we aimed to expand previous findings and investigate by computational docking method the homology, identity and similarities in the genome sequence and structure between two already sequenced genome variants (Spanish and British) of SARS-CoV-2 and the structure and alignment of the predictive spike. Understanding the differences between these two variants can help us to understand how each one behaves in its cellular interaction, which will, in turn, serve to shed light on how the virus may behave in the future with potential new variants.

## 2. Materials and Methods

The genome analysis was based on the so-called Spanish (the first detected in Spain) and British (alpha, lineage B.1.1.7) variants (Table 1). The Spanish variant analyzed was first detected and sequenced in Spain in February 2020, while the British variant was first detected and sequenced in the United Kingdom in September 2020. The full-length genome sequence was downloaded from the Global Initiative on Sharing All Influenza Data (GISAID; https://www.gisaid.org/ accessed on 30 October 2021).

### 2.1. Alignment of the Two Genomes

We evaluated the statistics of sequence identity and sequence similarity using the Sequence Identity And Similarity (SIAS) service provided by the Universidad Complutense de Madrid, Spain (http://imed.med.ucm.es/Tools/sias.html accessed on 30 October 2021). To calculate pairwise identity and similarity of a Multiple Sequence Alignment, we applied both the BLOSUM62 [9] and GONNET [10] methods.

We used a multiple alignment of nucleic acid and protein sequences software (Clustal X, Clustal, Dublin, Ireland) to calculate a Percent Identity Matrix (PIM) with the score obtained for each pair of aligned sequences, and to construct an automatic multiple alignment of nucleotide or amino acid sequences [11].

### 2.2. Translation of the Nucleotide Sequence into Aminoacidic Sequences

The translation of the peptide sequences from the nucleic acid sequences was estimated with the European Bioinformatics Institute’s European Molecular Biology Laboratory using EMBOSS Transeq [12]. One-letter notation of amino acid sequence was used [13].

### 2.3. Protein Structure Homology-Modelling, Simulation and Comparations

We used homology modelling for the construction of tertiary protein structures using the reference structures stored in the SWISS-MODEL (https://swissmodel.expasy.org/ accessed on 30 October 2021) [14]. The SWISS-MODEL server is a fully automated protein structure homology-modelling server. The tool builds the base structure in three dimensions following similarity parameters that have been analyzed and contrasted, with the appropriate acceptance criteria. Repositories are proposed by the program once all their existing homologies have been evaluated, with a minimum of 30% identity with the amino acid sequence of the protein. The SWISS-MODEL runs on the OpenStructure computational structural biology framework [15] and the ProMod3 modelling engine is needed to perform this step. A Global Model Quality Estimation (GMQE) is scored from zero to 1 and measures the quality of the model based on its alignment, with numbers closer to 1 indicating a more reliable model. Qualitative Model Energy Analysis (QMEAN) measures the quality of the model based on structural features and the quality of the chemistry, including beta carbon (Cβ) interaction, all-atom interaction, torsion angles and solvation as geometric evaluators. The more positive the score, the better the model, although a good model can have a negative QMEAN score. However, a score below −4 indicated a model with poor chemistry, which compromises the viability of the protein structures of the prediction. A Local Quality Estimate indicates the quality of the model on a per residue basis and can show if some sections of the model are problematic (such as the ends of the model in the report above). Furthermore, an extension of the single model composite score QMEAN can be obtained by applying a consensus-based distance constraint (DisCo) score. QMEANDisCo is implemented through a feed-forward neural network, thus improving the accuracy of the model prediction [16].

### 2.4. Antigenicity and Binding Receptor Domain Interaction with ACE2

We used PyMOL to visualize and compare the molecules under study and produce images [17]. This is an open-source, powerful molecular visualizer, which is extremely practical for working with proteins. PyMOL is suitable for producing high-quality 3D images of biological molecules, as well as allowing measurement of certain parameters, comparison between molecules and a few basic simulations. PyMOL also allows electrostatic calculations using the Adaptive Poisson-Boltzmann Solver plugin. This program interface permits us to make electrostatic calculations and visualize potential energy surfaces and charge densities on protein surfaces. We used this Adaptive Poisson-Boltzmann Solver plugin to predict protein interaction sites and also to map antigen epitopes [18].

### 2.5. Computational Prediction of Protein-Protein Docking Results

Finally, we performed the docking or in-silico prediction of the molecular coupling of protein–protein complex structures using the Rosetta Ligand Docking software [19]. This technique enables us to model molecular interactions between protein and other small molecules and helps us to understand the biological mechanisms of ligands and the therapeutic design of proteins [20].

## 3. Results

### 3.1. Alignment of the Two Genomes

**Figure 1:**
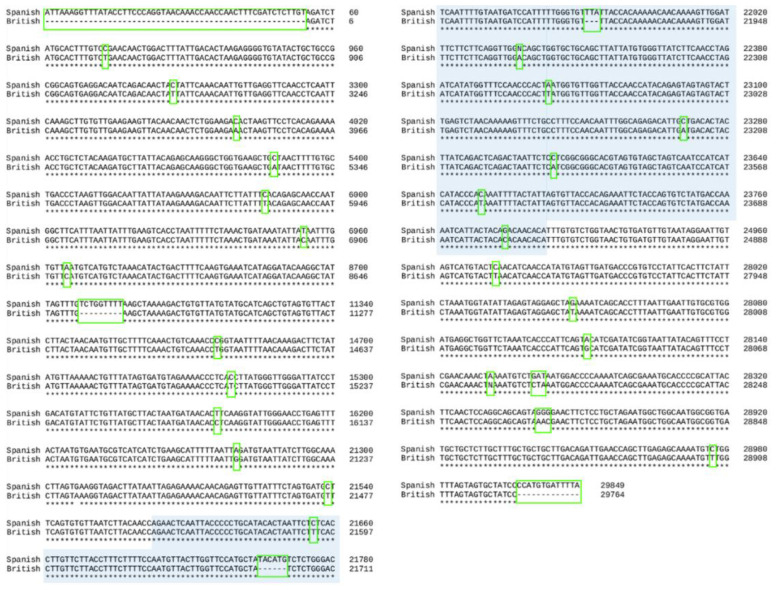
Complete genome alignment of Spanish and British variants. In light blue, spike gene alignment of the two variants. The differences between the two sequences are marked with a green box. A consensus line is shown below the nucleotide alignment of the two variants, with the following symbols indicating the degree of conservation observed for each compared pair: “*” (identical residues in all sequences).

The result of the alignment of the complete genome is shown in Figure 1. The alignment obtained produced four deletions, the largest one situated in the 5′ termination of the nucleotide sequence. There were 23 nucleotide substitutions also predicted which could affect the functionality of the proteins produced from this sequence. However, the similarity sequence scored resulted in 99.99 similarity between the two variants, which suggests that the genome identity between these two variants is almost perfect. The identity results between the Spanish and British SARS-CoV-2 variants were 28.67%, and the PIM calculated between the two variants showed that the number of identical residues after alignment was very high (99.88%).

The alignment obtained for the spike protein gene resulted in a deletion near to the N- terminus and 10 substitutions (Figure 1). Despite these mutations, the similarity sequence for the identity between the genes from the two variants resulted in a 99.97 similarity, which suggests that the genome identity encoding the S protein between these two variants is almost perfect. The identity results of the complete genome between Spanish and British variants were 32.46%, and the PIM calculated between the two SARS-CoV-2 variants showed that the number of identical residues after alignment was very high (99.76%)

### 3.2. Translation of the Nucleotide Sequence into Aminoacidic Sequences

**Figure 2:**
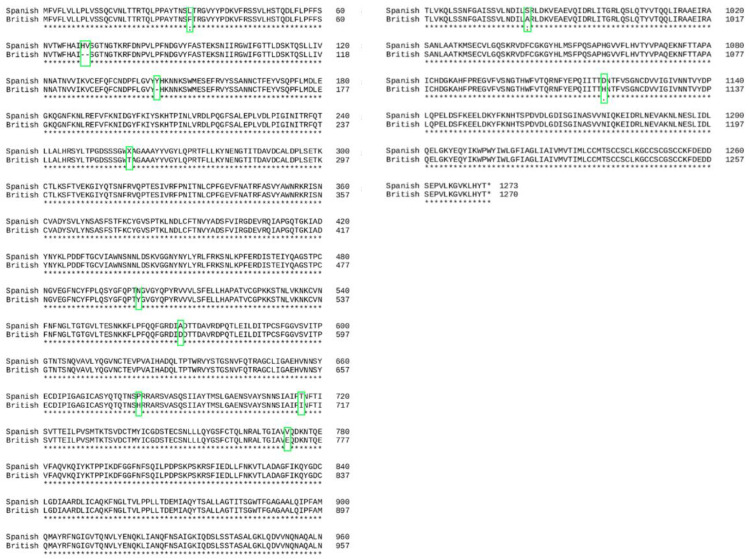
Translation of the spike gen into amino acid sequence in the two variants. A consensus line is shown below the amino acid alignment of the two variants, with the following symbols indicating the degree of conservation observed for each compared pair: “*” (identical residues in all sequences), “:” (highly preserved column), “.” (weakly preserved column). One-letter notation of amino acid sequence used from reference [13].

Figure 2 shows the result of the translation of the aligned nucleotide sequences which encode the proteins of the two variants under study into their corresponding aminoacidic sequences. The study of the two sequences resulted in 3 amino acid deletions and 9 amino acid changes.

### 3.3. Protein Structure Homology-Modelling, Simulation and Comparations

**Figure 3:**
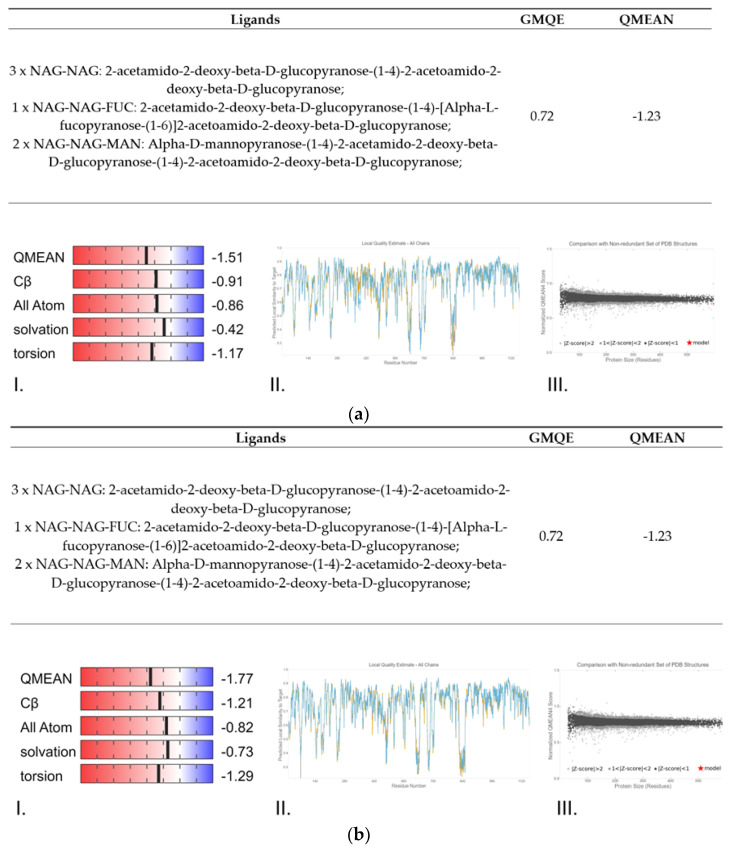
Protein Structure Homology-Modelling of the spike for (**a**) Spanish variant and (**b**) British variant. Subsections: I. QMEAN Z-Scores. II. QMEANDisCo Local. III. Protein size. Model-Template alignment shows how well template structure and sequence align and what parts of the model were used. Better alignment is marked in blue, worse alignment and modeling in red. Secondary structure is also indicated with tubes for α-helix and arrows for β-sheet. Both QMEANs scores (−1.23 and −1.25) show extremely high model quality.

**Figure 4:**
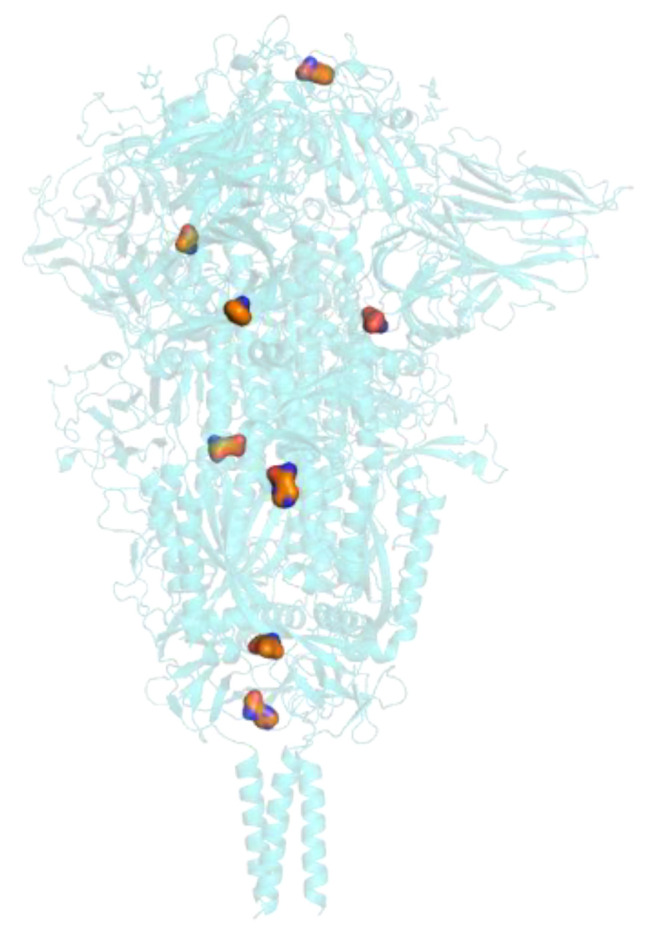
Spike structure of outstanding mutations of Spanish and British SARS-CoV-2 variants.

The protein modelling of the spike of both variants resulted in the prediction shown in Figure 3, which shows the model quality analysis obtained with SWISS-MODEL. Taking all this predicted data into consideration, we can affirm that there are minimal structural differences between the Spanish and British variants. After studying the conformation of the spike from both variants, a similar structure was produced, with few notable changes between the two variants (Figure 4).

### 3.4. Antigenicity

**Figure 5:**
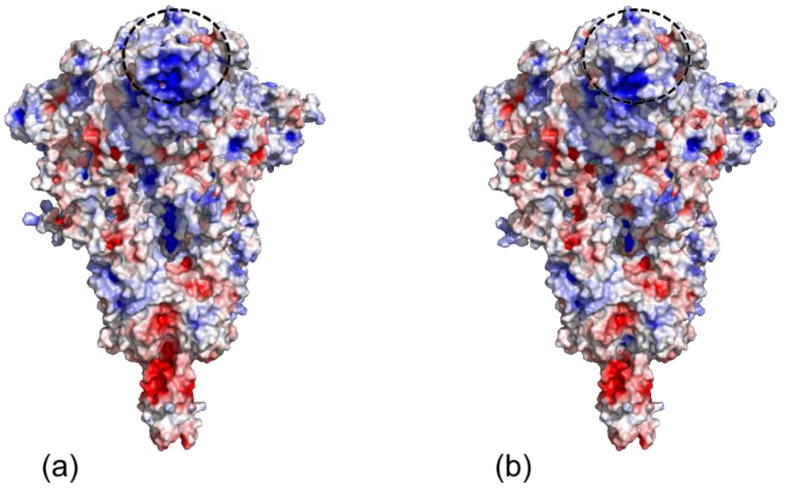
Comparison of antigenicity of SARS-CoV-2 Spanish and British variants. (**a**) Spanish variant. (**b**) British variant. By convention, red is negative and blue positive. Change in antigenicity indicated by the dotted circle.

Figure 5 shows the predicted spike antigenicity from both variants, which was higher in the British variant than the Spanish one, based on the prediction of the antigenicity with protein surfaces by consensus scoring in PYMOL.

### 3.5. Binding Receptor Domain Interaction with ACE2

**Figure 6:**
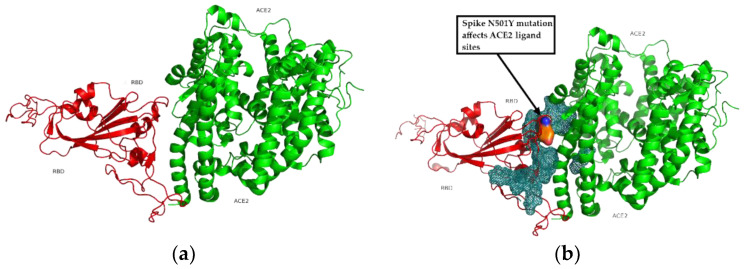
Interaction between binding receptor domain from spike protein and ACE2 receptor. (**a**) Spanish variant. (**b**) British variant.

The interaction between the binding receptor domain from the spike protein and the *ACE2* receptor is plotted and the bond between them is simulated in Figure 6. As can be seen, the ligand zone between *ACE2* (a full agonist of SARS-CoV-2) and the Binding Receptor Domain of the virus, compromises some of the mutations found and the energy of this ligand therefore varies. The spike N501Y mutation in the British variant affects *ACE2* ligand sites.

### 3.6. Computational Prediction of Protein-Protein Docking Results

**Figure 7:**
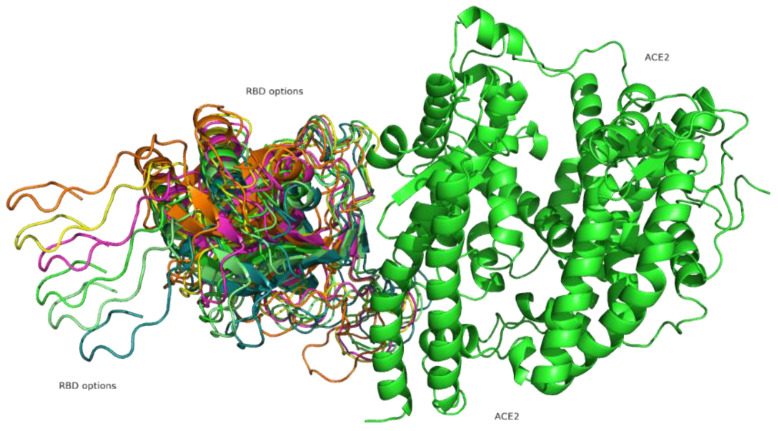
Computational Prediction of binding receptor domain (BRD) from the spike protein and the ACE2 receptor, showing all possible interactions.

Figure 7 shows the result of the protein-protein docking algorithm. All the possible bonds between the proteins are presented in different colours, showing several predicted binding options.

Receptor binding domain (RBD) from the spike protein is shown in red. The binding region in the mesh surface of the British variant bound to the ACE2 receptor shown in orange, with British mutations highlighted in surface display.

## 4. Discussion

The present analysis shows that the identity obtained between the Spanish and British variants is low and the similarity of the whole genome and the spike is extremely high. In addition, the antigenic neutralization capacity of the British variant has decreased. Our results indicate that minimal mutations could interfere in the infectiveness of the virus due to the change of the fitness between the host cell recognition and interaction proteins. A N501Y substitution is situated in the RBD of the spike of the British variant, which may be the reason for its extraordinary potential for infection.

SARS-CoV-2 is a highly contagious virus that infects humans and produces a respiratory distress syndrome, which has led to a global pandemic and a major crisis in most countries around the world. The present study provides a picture of the end point structure of the RBD-ACE2 binding event, and the conformation and dynamics between both molecules in the two variants. These variants contain a number of mutations that confer enhanced affinity for ACE2 and lend the virus competitive advantages in terms of infection, spread and immunity from neutralizing antibodies.

Spike protein is responsible for initiating the infection by linking with host cell receptors and is known to be responsible for everything related with host cell interaction and cell membrane fusion (virus-host) at the outset of the infection. It splits into two subunits (S1 and S2) during infection. Domain S1 contains the region which facilitates the union with the first receptor, while S2 controls membrane fusion. This process is mediated by the bond between the RBD and the Internal Fusion Peptide and the host membrane receptors [21,22]. The spike consists of a trimer that has two basic prefusion conformations: up/closed and down/open. When one of the peak monomers is deployed in its prefusion conformation, the process of binding with the first receptor of the host cell begins. Therefore, SARS-CoV-2 is transported into the cell, enters the cytosol and replicates by binding to ACE2 receptors on the cell membrane [23,24]. Understanding the differences between the different variants is key in the fight to control this pandemic, for two reasons: firstly, the new variants may have greater infectivity and transmissibility, which contributes to the spread of the virus; in addition, although this seems not to be the case with the British variant [25], the efficacy of vaccines could be compromised by a new variant [26].

Considerable efforts have therefore been made to characterize the interaction between the Spike protein RBD and the human ACE2 receptor. To date, over 5700 genome variants have been identified [27], with differences in the analytical data from different parts of the world [28]. The fact that some mutations have been acquired by different independent lineages in separate countries suggests there is a strong positive selection and possibly reflects an adaptive evolution of the virus in response to either the epidemiological control measures or a growing immunity to the original viral variants. Positive selection could therefore favor increased transmissibility, infectivity and/or immune escape of the virus [29].

Previous studies have shown that, by creating single amino acid substitution mutations in the SARS-CoV-2 sequences, SARS-CoV-2 can use two types of amino acid residues to keep up its binding activity with ACE2 [30]. The receptor binding capacity is enhanced by certain changes, such as by introducing amino acid changes at N501, P499, Q493, F486, A475 and L455, while it is decreased by replacing residues Q498, E484, T470, K452 and R4391. Indeed, the spike of the SARS-CoV-2 British variant includes one of these key replaced residues in the receptor binding motif that interacts with ACE2 and the neutralizing antibodies. This specific mutation is N501Y, and this may be the reason for the extremely infective potential of this variant. Therefore, the docking trial resulted in several possible bindings between this mutated spike and the ACE2 receptor.

The British variant, B.1.1.7, of SARS-CoV-2, which is reported in the GISAID gene bank with the accession EPI_ISL_877562, includes the following mutations from the original Wuhan sequence: Spike A570D, Spike D614G, Spike D1118H, Spike N501Y, Spike P681H, Spike Q52K, Spike S982A and Spike T716I, while there is also a gap of 16 nucleotides when compared to the Spanish one. This variant is more transmissible than the previously circulating viruses, with an estimated increase of between 50% and 74% in transmissibility, thus adding 0.4 to the basic reproduction number R0, bringing it to a range of 1.5 to 1.7 [31]. Previous studies of molecular dynamics simulations have found novel unbound substrates that may represent new targets for therapeutic design [32].

Some previous studies have evaluated different mutations in the spike protein of SARS-CoV-2. A recent article identified N501Y as vital for infectivity and indicated that there are other mutations that open a potentially therapeutic aim at the interface between spike glycoprotein subunits S1 and S2 [6]. On the other hand, the work by Villoutreix et al. is also an in silico model suggesting that the N501Y mutation would be enough to compensate for two other substitutions of K417N and E484K of the South African strain that would appear neutral or even unfavorable [7]. Finally, Liu et al. in a preprint publication used an animal model and cellular cultures to suggest a potential role of N510Y as a major adaptive spike mutation of major concern [8]. The present study completes the previous studies of N501Y in SARS-CoV-2 strengthening the importance of this variant. Interestingly, in both variants, there is a very relevant mutation D614G, which fundamentally affects the interlocking of pertussis and therefore the ability to cause fusion between the cell and viral membranes. However, this being very relevant, in the British variant the determining mutation that affected infectivity was N501Y. It is also a remarkable fact that the mutation that generates the new class, and identifies the new variant is not the N501Y but the E484K.

Different studies have evaluated the different aspects of mutations in the spike protein of SARS-CoV-2 when comparing the British and South African strains [33]. Antigenicity is a key property of a viral protein in order to be recognized and neutralized by the immunogenic system. Indeed, small mutations, including hidden amino acids, could interfere in the binding affinity with different inhibitory abilities of the interaction between the spike protein and the ACE2 receptor [34]. While a number of vaccines and monoclonal antibodies have been developed extremely successfully and quickly and are currently in use, numerous variants of the causative SARS-CoV-2 are emerging [28,35] and threatening the efficacy of vaccines and monoclonal antibodies [36]. Interestingly, these escape mutations, which were rare in the first wave, together with rebound outbreaks, have accelerated the appearance of new mutations, which influences the long-term endemic level in a combined effect between infection derivation and vaccine implementation [37,38]. Accordingly, new methods have emerged to quickly detect new variants of concern from regular strains of SARS-CoV-2 [39].

In conclusion, this study of the comparison of the sequences and structure of SARS-CoV-2 Spanish and British variants, focusing on the spike, indicates that the identity is low and the similarity of the whole genome and the spike is extremely high. The structure of the predictive spike is almost identical, despite occasional mutations. However, the British variant has a decreased antigenic neutralization capacity in the distal zone of the spike protein, which makes it more difficult for the immune system to recognize it and create antibodies against it. The N501Y substitution, situated in the RBD of the spike of the British variant, might be the reason for its extraordinary infective potential.

## Figures and Tables

**Table 1:** Details of the two SARS-CoV-2 variants.

	Spanish Variant	British Variant
Virus name	hCoV-19/Spain/CN-ISCIII-201048/2020	hCoV-19/England/MILK-9E05B3/2020
Accession ID	EPI_ISL_539531	EPI_ISL_601443
Type	Betacoronavirus	Betacoronavirus
GISAID Clade	G	GR
Lineage	B.1	B.1.1.7
Mutations (AA)	Spike D614G, Spike E773V, Spike F32L, NSP3 A358V, NSP3 E427D, NSP4 H31N, NSP12 P323L, NSP16 G208R, NSP16 K277E, NSP16 V294A	Spike A570D, Spike D614G, Spike D1118H, Spike H69del, Spike N501Y, Spike P681H, Spike S982A, Spike T716I, Spike V70del, Spike Y145del, N D3L, N G204R, N R203K, N S235F, NS8 Q27stop, NS8 R52I, NS8 Y73C, NSP3 A890D, NSP3 I1412T, NSP3 T183I, NSP6 F108del, NSP6 G107del, NSP6 S106del, NSP12 P323L
Collection date	20 February 2020	20 September 2020
Location	Europe/Spain/Canary Islands	Europe/United Kingdom/England
Host	Human	Human
Originating laboratory	Hospital Universitario Nuestra Señora de Candelaria, Tenerife, Spain	Lighthouse Lab, Milton Keynes, United Kingdom
Submitting laboratory	Instituto de Salud Carlos III, Madrid, Spain	Wellcome Sanger Institute for the COVID-19 Genomics UK (COG-UK) consortium

## Data Availability

All the data are included in the present manuscript.
